# Sensitivity of two methods to detect *Mycoplasma agalactiae* in goat milk

**DOI:** 10.1186/s13620-015-0049-y

**Published:** 2015-09-07

**Authors:** J. Tatay-Dualde, A. Sánchez, M. Prats-van der Ham, A. Gómez-Martín, A. Paterna, J.C. Corrales, C. de la Fe, A. Contreras, J. Amores

**Affiliations:** Research Group on Ruminant Health, Animal Health Department, School of Veterinary Sciences, Regional Campus of International Excellence “Campus Mare Nostrum”, University of Murcia, 30071 Murcia, Spain

**Keywords:** Goat, Contagious agalactia, PCR, Culture, Milk, *Mycoplasma agalactiae*

## Abstract

**Background:**

Laboratory diagnostic techniques able to detect *Mycoplasma agalactiae* are essential in contagious agalactia in dairy goats. This study was designed: 1) to determine the detection limits of PCR and culture in goat milk samples, 2) to examine the effects of experimental conditions including the DNA extraction method, PCR technique and storage conditions (fresh *versus* frozen stored milk samples) on these methods and 3), to establish agreement between PCR and culture techniques using milk samples from goats with mastitis in commercial dairy herds. The study was conducted both on artificially inoculated and field samples.

**Results:**

Our findings indicate that culture is able to detect *M. agalactiae* in goat milk at lower concentrations than PCR. Qualitative detection of *M.agalactiae* by culture and PCR was not affected by sample freezing, though the DNA extraction method used significantly affected the results of the different PCR protocols. When clinical samples were used, both techniques showed good agreement.

**Conclusions:**

The results from this study indicate that both culture and PCR are able to detect *M. agalactiae* in clinical goat mastitis samples. However, in bulk tank milk samples with presumably lower *M. agalactiae* concentrations, culture is recommended within the first 24 h of sample collection due to its lower limit of detection. To improve the diagnostic sensitivity of PCR in milk samples, there is a need to increase the efficiency of extracting DNA from milk samples using protocols including a previous step of enzymatic digestion.

## Background

Contagious agalactia (CA) affects small ruminants and may cause a triad of clinical symptoms comprising keratoconjunctivitis, polyarthritis and mastitis. In addition, the syndrome may also produce respiratory and reproductive symptoms in goats. The disease is caused by several bacteria of the genus Mycoplasma although *M. agalactiae* is its main causative agent [1].

To determine the epidemiological situation of goat herds in endemic areas of contagious agalactia (CA), systematic diagnostic studies carried out on milk samples are strongly recommended [2]. There are two kinds of diagnostic methods used to detect infected herds: indirect serological detection, exclusively when animals are not vaccinated, and direct evidence of *M. agalactiae* in milk by means of culture and/or PCR [1]. In these studies, bacteriological techniques need to be conducted on milk samples in order to specifically detect the presence of CA causative agents. Therefore, particular media such as Eaton, Hayflick, pH or SP4II are recommended [3]. PCR protocols designed for the identification of the mycoplasma species involved in caprine CA offer more rapid results and improved sensitivity [4–6]. However, DNA extraction and purification methods have also been considered to play a major role in the obteining of PCR results [7]. In this sense, Tola et al. [8] developed a method to extract DNA directly from milk samples, and nowadays some commercial kits are also available for this purpose.

The aim of this study was to determine the lower limits of detection of culture and PCR techniques for *M. agalactiae* in experimentally inoculated goat milk samples. We also examined the effects of sample freezing and the DNA extraction method used on these techniques. In a second experiment, agreement between PCR and culture techniques was assessed using milk samples from mastitic goats from dairy herds of a CA endemic area.

## Methods

For the experimental study, five goat milk samples were obtained: one bulk tank milk sample and four from randomly selected goats. All samples tested negative for *M. agalactiae* by conventional culture [9] and PCR [5].

Each of these five samples was divided in seven 4 ml-aliquots which were inoculated with serial fold dilutions in the range 10 to 10^7^ (except one of the samples, which yielded a concentration of 10^6^) CFU/ml of a PG2 inoculum (reference strain of *M. agalactiae* NCTC 10123). In addition, a negative control (non inoculated aliquot) was included for each sample. Then, each aliquot was divided into two 2 ml aliquots: one was examined after 24 h of refrigerated storage and the other after freezing (−21 °C) for 14 days and thawing at room temperature. A total of 80 aliquots were analyzed. 200 μl of the samples were cultured in liquid PH medium [10]. After 24 h of incubation at 37 °C, the samples were filtered (0.45 μm), and afterwards cultured in agar PH medium so as to detect the presence of typical mycoplasma colonies. Two further 200 μl-aliquots were obtained to use two different DNA extraction methods, one as described by Tola et al. [8] and the other using a commercial kit (High Pure PCR Template Preparation Kit, Roche Diagnostics). Subsequently, all samples were analyzed using the three PCR protocols previously described [4–6]. PCR conditions are detailed in Table 1. PCR results were compared with a negative control and a positive control (DNA of the PG2 strain).

**Table 1:** PRC conditions for each protocol

Reference	Primers	PCR conditions
Tola et al. (1996) [8]	5′-AAAGGTGCTTGAGAAATGGC-3′	94 °C, 5 min.; (94 °C, 1 min. 64 °C, 1 min.; 72 °C,1 min) x 35 cycles; 72 °C, 10 min.
	5′-GTTGCAGAAGAAAGTCCAATCA-3′	
Marenda et al. (2005) [5]	5′-CATTGAACCTCTTATGTCATTTACTTTG-3′	94 °C, 5 min.; (94 °C, 1 min.; 58 °C, 1 min.; 72 °C, 1 min.) x 30 cycles; 72 °C, 10 min.
	5′-CTATGTCATCAGCTTTTGGGTGA-3′	
De la Fe et al. (2012) [6]	5′-GCAGCTTGTTTAGTGTCAAAG-3′	94 °C, 2 min.; (94 °C, 30 sec.; 49 °C, 30 sec,; 72 °C, 30 sec.) x 30 cycles; 72 °C, 5 min.
	5′-CCTAAAGCAACCTTTATAACTG-3′	

For the field study, 255 clinical mastitis samples were obtained from 80 dairy goats herds reared in an endemic CA area. All samples collected were kept refrigerated at 4 °C and arrived at our laboratory within the following 24 h. Immediately after their arrival, these samples were cultured following the same protocol described in our experimental study. Then, 200 μl of each milk sample were processed with DNA extraction prior to PCR diagnosis [8, 5].

Agreement between PCR and culture techniques was determined using Win Episcope 2.0 software [11]. Thursfield criteria [12] were used to interpret kappa values. The Chi squared test implemented in Epi Info 3.5.2 software [13] was used to assess the factors that affect the sensitivity of the techniques, which was calculated as the proportion of inoculated samples detected by the different methods used.

## Results and discussion

The PCR protocols described by Tola et al*.* [4], Marenda et al*.* [5] and De la Fe et al*.* [6] did not differ significantly from each other. Moreover, no significant differences were detected between fresh and frozen samples. When comparing the DNA extraction methods used together with the PCR protocols, the commercial kit allowed the detection of 44.6 % positive samples (95 % CI; 37.8 %, 51.4 %), while this rate was significantly lower, 33.8 %, after using the extraction method described by Tola et al*.* [8] (95 % CI; 27.3 %, 40.3 %) (Table 2). Negative controls showed negative results in all cases. These differences observed between the DNA extraction methods could be explained by the additional step of enzymatic protein denaturation carried out by proteinase-K when using the commercial kit. Furthermore, it has been demonstrated that the use of this enzyme could also inactivate some inhibitors which are present in milk samples [14]. In this sense, Becker et al*.* [7] obtained a lower limit of detection of mycoplasmas when dilutions were prepared in water rather than milk.

**Table 2:** PCR results obtained according to the DNA extraction method and the *Mycoplasma agalactiae* concentration used

*M. agalactiae* concentration (CFU/ml)	Method of extraction					
	Tola et al*.* (1997) [8]			Commercial kit^c^		
	Negative	Positive	Se (%), 95 % CI	Negative	Positive	Se (%), 95 % CI
10^1^	30	0	0	30	0	0
10^2^	30	0	0	29	1	3.3, (−3.1 %, 9.8 %)
10^3^	30	0	0	24	6	20, (5.7 %, 34.3 %)
10^4^	28	2	6,7, (−2.3 %, 15.6 %)	22	8	26.7, (10.8 %, 42.5 %)
10^5^	15	15	50, (32.1 %, 67.9 %)	8	22	73.3, (57.5 %, 89.2 %)
10^6^	2	28	93,3, (84.4 %, 102.3 %)	0	30	100
10^7^	0	24	100	0	24	100
Total	135	69	33.8,^a^ (37.8 %, 51.4 %)	113	91	44.6,^b^ (27.3 %, 40.3 %)

*Se* sensitivity at each concentration

^a, b^: Values with different superscripts differ significantly (*p* < 0.05)

^c^: High Pure PCR Template Preparation Kit, Roche Diagnostics

The detection limit yielded was lower for the culture technique than for the PCR protocols (10 CFU/ml with a sensitivity of 33.3 %) (Table 3), and there were no significant differences between fresh and frozen samples. Thereby, the culture method was able to detect more positive samples than the PCR protocols used and its detection limit was also lower, suggesting a lower sensitivity of PCR when used directly in milk samples.

**Table 3:** Culture results according to the concentration of *Mycoplasma agalactiae* used to inoculate the goat milk samples

*M. agalactiae* concentration (CFU/ml)	Culture results		
	Negative	Positive	Sensitivity (%)
10^1^	38	18	33.3
10^2^	12	42	77.8
10^3^	12	42	77.8
10^4^	6	48	88.9
10^5^	0	54	100
10^6^	0	54	100
10^7^	0	42	100

In previous studies, it has been observed that *M. agalactiae* can be found in milk at concentrations of 10^6^ up to 10^8^ CFU/ml, [15]. Moreover, Castro-Alonso et al*.,* [16] showed by experimental inoculation that this concentration could even reach values of 10^10^ to 10^12^ CFU/ml. Therefore, considering our limits of detection (above 10^6^ with culture and PCR), both PCR and culture methods should be able to detect the presence of mycoplasmas from the initial days postinfection when the concentrations of *M. agalactiae* are above 10^6^ CFU/ml, because both techniques show high values of sensitivity [15, 16]. However, when the concentration of mycoplasmas is unknown, as it happens with the bulk tank milk samples, where obviously these values are lower than in individual samples from infected animals, the use of PCR protocols for bulk tank samples with a limit of detection of 10^2^–10^4^ could be inadequate. Therefore, for the diagnosis of CA from bulk tank milk, samples should be previously cultivated and the DNA extraction should be carried out on the culture rather than on milk sample, in order to improve its sensitivity [17].

Of the 255 clinical samples examined, 58 were culture positive and 47 were PCR positive for *M. agalactiae*, while 41 samples were culture and PCR positive and 191 scored negative using both techniques. Our agreement study revealed a kappa coefficient of 0.725, indicating a good agreement between the two methods [12]. This is probably due to the concentration range of *M. agalactiae* which is usually found in mastitic goat milk samples (10^6^ to 10^12^ CFU/ml), that has been previously described [15, 16].

## Conclusion

In conclusion, our findings show that both culture and PCR are able to detect *M. agalactiae* in clinical goat mastitis samples. However, in bulk tank milk samples with presumably lower *M. agalactiae* concentrations, culture within the first 24 h of sample collection is recommended. In order to improve the diagnostic sensitivity of PCR in milk samples, it is also necessary to increase the efficiency of the DNA extracting methods from milk samples, for example by using protocols with a previous enzymatic digestion so as to reduce the presence of inhibitors in our sample.
